# Characteristics of Community-Associated Methicillin-Resistant *Staphylococcus aureus* (CA-MRSA) Strains Isolated from Skin and Soft-Tissue Infections in Uruguay

**DOI:** 10.1155/2009/472126

**Published:** 2009-10-22

**Authors:** Lorena Pardo, Virginia Machado, Marta Mollerach, María Inés Mota, Lorena P. N. Tuchscherr, Pilar Gadea, Noella Gardella, Daniel O. Sordelli, Magdalena Vola, Felipe Schelotto, Gustavo Varela

**Affiliations:** ^1^Department of Bacteriology and Virology, Institute of Hygiene, School of Medicine, Universidad de la República, Alfredo Navarro, 3051 Montevideo, Uruguay; ^2^Cátedra de Microbiología, Facultad de Farmacia y Bioquímica, Universidad de Buenos Aires, Junín 956, 1113 Buenos Aires, Argentina; ^3^Department of Microbiology, School of Medicine, University of Buenos Aires, 1113 Buenos Aires, Argentina

## Abstract

We analyzed 90 nonduplicates community-associated methicillin-resistant *S. aureus* (CA-MRSA) strains isolated from skin and soft-tissue infections. All strains were *mecA* positive. Twenty-four of the 90 strains showed inducible macrolide-lincosamide-streptogramin B resistance. All strains produced *α*-toxin; 96% and 100% of them displayed positive results for *lukS-F* and *cna* genes, respectively. Eigthy-five strains expressed capsular polysaccharide serotype 8. Six different pulsotypes were discriminated by pulsed-field gel electrophoresis (PFGE) and three predominant groups of CA-MRSA strains (1, 2, and 4) were identified, in agreement with phenotypic and genotypic characteristics. Strains of group 1 (pulsotype A, CP8+, and Panton-Valentine leukocidin (PVL)+) were the most frequently recovered and exhibited a PFGE band pattern identical to other CA-MRSA strains previously isolated in Uruguay and Brazil. Three years after the first local CA-MRSA report, these strains are still producing skin and soft-tissue infections demonstrating the stability over time of this community-associated emerging pathogen.

## 1. Introduction

*Staphylococcus aureus *is capable of establishing a wide spectrum of interactions with the human host. It can be part of the microbial flora or cause a variety of illnesses ranging from mild infections compromising skin and soft-tissues to severe life-threatening diseases such as necrotizing pneumonia, bacteremia, osteomyelitis, toxic shock syndrome, and meningitis [1]. *S. aureus *harbors several virulence factors including surface-associated adhesins, secreted exo-proteins and toxins [2–5]. Another important characteristic of *S. aureus* is the capacity to acquire resistance to antimicrobial agents. The first isolation of methicillin-resistant *S. aureus* (MRSA) was reported in 1960 and since then the prevalence of MRSA has increased in all scrutinized regions, with different figures even within the same country [6, 7]. Methicillin resistance is conferred by the *mecA* gene which codes for an additional penicillin-binding protein, namely, 2a (PBP2a) with reduced affinity to *β*-lactam agents. This gene is located in a mobile genetic element of variable size called staphylococcal cassette chromosome *mec* (SCC*mec*). So far, seven types and several subtypes of SCC*mec* have been characterized [8–11].

 Since 1990 an increasing number of cases produced by MRSA acquired in the community have been reported, especially in children and young adults without the classical risk factors involved in healthcare-associated methicillin-resistant *S. aureus *(HA-MRSA) infections [12–15]. These strains were called CA-MRSA (community-associated methicillin-resistant *S. aureus*) to distinguish them from HA-MRSA. Generally HA-MRSA strains are resistant to other antibiotics different from *β*-lactams, whereas CA-MRSA are mostly resistant to methicillin only. HA-MRSA isolates frequently harbor SSC*mec* types-I, II and III whereas CA-MRSA strains carry types IV, V, VI and VII. Finally, unlike HA-MRSA, the majority of CA-MRSA strains carry the* lukS-F* genes which code for the Panton-Valentine leukocidin (PVL) [9, 16].

 Previously, Ma et al. [17] reported the results of a CA-MRSA outbreak that took place in Uruguay between 2002-2003. The cases informed in this study were observed in children, young adults and inmates. Boils and abscesses were the most prevalent infections, followed by hidradenitis and cellulitis.

 The aim of this study was to establish the phenotypic and genotypic characteristics of CA-MRSA strains recovered from skin and soft-tissues infections.

## 2. Materials and Methods

We analyzed 213 *S. aureus* strains obtained between December 2004 and November 2005 from 213 outpatients with skin and soft-tissue infections (SSTI). These strains were recovered from four different laboratories, three of them located in Montevideo and the metropolitan area and the other located 400 Km from Montevideo.

 Clinically and epidemiologically relevant information from each patient were collected from the medical records and patient interviews. Susceptibility to antimicrobial agents was determined by the disk diffusion method in accordance with the Clinical and Laboratory Standards Institute guidelines [18]. The antibiotics tested included oxacillin, cefoxitin, ciprofloxacin, tetracycline, gentamicin, rifampin, trimethoprim/sulfamethoxazole, chloramphenicol, vancomycin, erythromycin, and clindamycin (Oxoid Ltd., Basingstoke, Hampshire, UK). *S. aureus* strain ATCC 25923 was used as quality control. The double disk diffusion test was performed to determine inducible clindamycin resistance (iMLS_B_) [18].

 Susceptibility to mupirocin and fusidic acid was also tested by the disk diffusion method. Results were interpreted according to Fuchs et al*.* [19] and to the French standards for mupirocin and fusidic acid, respectively [20].

 MICs to oxacillin and vancomycin were determined by the agar dilution method [18].

 Ninety out of the 213 strains were defined as CA-MRSA based on the epidemiologic analysis of the patients [21] and their antibiotic susceptibility profile: cefoxitin-resistant and susceptible to trimethoprim/sulfamethoxazole, ciprofloxacin, tetracycline and gentamicin. These 90 CA-MRSAstrains were further analyzed as described in what follows.

### 2.1. Virulence Factor Identification

Alpha-hemolysin was detected in accordance with the procedure described by Cooper et al. [22] *S. aureus* Wood 46 strain was included as a positive control. The presence of *β*-hemolysin was ascertained by demonstration of increased hemolysis in sheep blood agar plates incubated at 37°C for 24 hours and then at 4°C for other 24 hours (heat-cold lysis). CP typing was performed by colony immunoblot method with CP5 and CP8-specific antibodies as described by Lee et al. [23]. Strains showing a negative reaction to this test were later investigated by immunodiffusion. Each strain was tested at least twice and those isolates with no reaction to CP5 and CP8 antibodies were defined as nontypeable (NT). The presence of *cap5* or *cap8* genes in NT strains was determined by PCR amplification as described elsewhere [24].

### 2.2. Genotyping

Bacterial DNA was obtained from isolated colonies using the Wizard genomic DNA preparation kit (Promega. Madison, Wis, USA) adding 20 mg/mL lysostaphin (Sigma Chemical) in the cell-lysis step [25]. The presence of *cna, mecA* and *lukS*-*lukF* genes was determined by PCR amplification [26–28].

 SCC*mec* typing was performed by a multiplex PCR method only on 23 isolates belonging to group1 and on four group 4 strains (described in what follows) [10].

### 2.3. Pulsed-Field Gel Electrophoresis (PFGE)

DNA macrorestriction and separation of fragments by PFGE were conducted using standardized procedures [29]. DNA was digested with SmaI (New England Biolabs). PFGE conditions were 6 V/cm at 11,3°C for 23 hours, with pulses of 5 to 35 seconds. Electrophoresis was performed using a CHEF DR II instrument (Bio-Rad, Hercules, Calif, USA). Band patterns were visually interpreted following the criteria of Tenover et al. [30].

## 3. Results and Discussion

The* mecA* gene was detected in the 90 CA-MRSA strains analyzed. Nevertheless*, *3 out of the 90 strains displayed oxacillin inhibition zone diameters ≥13 mm. These results were similar to those described previously by other authors regarding the best performance of cefoxitin 30 *μ*g disk versus oxacillin 1 *μ*g disk for the screening of the MRSA phenotype confered by the *mecA* gene [31, 32]. The MIC for oxacillin of all the CA-MRSA strains ranged from 4 to 32 *μ*g/mL with a MIC_90_ of 16 *μ*g/mL, which is consistent with the heterogeneous resistance phenotype. These findings agree with reports showing that oxacillin MIC of CA-MRSA strains are lower than the values observed for HA-MRSA isolates [33].

 Twenty-four out of 90 (26.6%) CA-MRSA strains showed inducible macrolide-lincosamide-streptogramin B (iMLS_B_) resistance by the double-disk diffusion test. This figure is similar to that obtained by Patel et al. [34] and, as suggested by these authors, it might be due to the predominance of the cassette IV in Uruguayan CA-MRSA strains [17] which do not have the *erm* genes associated with inducible MLS_B_ phenotype. In this regard, twenty-three CA-MRSA strains belonging to group 1 (described in what follows) showed SSC*mec *type-IV, whereas four group 4 strains carried SCC*mec *elements type-II.

 Two isolates were resistant to chloramphenicol and rifampin. Rifampin has a potent antistaphylococcal activity, however resistance develops invariably when it is used as monotherapy for the treatment of *S. aureus* infections. Three of the 90 strains were resistant to mupirocin whereas all strains were susceptible to fusidic acid. These results suggest that fusidic acid could be used as a topical empiric adjuvant treatment for nasal eradication of CA-MRSA.

 All of the CA-MRSA strains studied were susceptible to vancomycin by the disk diffusion test and displayed MICs of ≤2 *μ*g/mL.

 All the CA-MRSA isolates produced *α*-hemolysin but none produced *β*-hemolysin.

 Eighty-five isolates expressed CP8, one was CP5-positive and four strains were NT. Those four NT isolates carried the *cap8* genes. This low prevalence of CP5 among the CA-MRSA (1%) could explain, at least partially, the absence of fatal cases observed in this group of patients [35].

Ninety-six percent of the CA-MRSA strains exhibited positive PCR amplification with *lukS-F *gene primers and all strains yielded positive amplification results with the *cna *gene primers. PVL production has been associated with CA-MRSA strains isolated from individuals with skin infections and necrotizing pneumonia [28, 36]. In contrast to CA-MRSA, HA-MRSA strains generally do not harbor the *lukS-lukF* genes. Therefore, the presence of the PVL genes emerged as a good local marker of CA-MRSA. With regard to the *cna* gene, several reports have described an association between the presence of this gene and the development of bacteremia associated with deep tissue infection [26, 37]. In this study we did not perform blood culture follow-up of the patients infected with CA-MRSA strains and there is no local information on the evolution of those infections caused by *cna* negative *S. aureus* strains.

**Figure 1:**
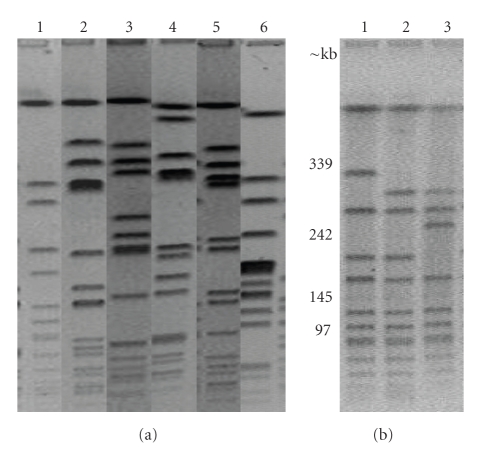
(a) Pulsed-field gel electrophoresis (PFGE) patterns of the SmaI-digested genomic DNA obtained from CA-MRSA isolates in Uruguay. Lane 1, pulsotype A (strain IH 23); lane 2, pulsotype B (strain IH 48); lane 3, pulsotype C (strain IH 46); lane 4, pulsotype D (strain IH 7); lane 5, pulsotype E (strain IH 22) and lane 6 pulsotype F (strain IH 69). (b) Pulsed-field gel electrophoresis (PFGE) patterns of the SmaI-digested genomic DNA obtained from CA-MRSA isolates in Uruguay. Lane 1, pulsotype A1 (strain IH 36); lane 2, pulsotype A (strain IH 44); lane 3, pulsotype A2 (strain IH 9).

 Six different pulsotypes (A–F) (Figure 1(a)) were identified by PFGE. Eigthy-two of the 90 strains belonged to the pulsotype A group (Figure 1(a), lane 1 and Table 1); 4 isolates were included in the pulsotype B, whereas C, D, E, and F pulsotypes were represented by one isolate each. Three minor subtypes within pulsotype A (A1, A2, and A3) (Figure 1(b)) were identified according to Tenove's criteria. Pulsotype A isolates exhibited a band pattern identical to those isolates identified by Ma et al. [17], being the most frequently found in Uruguay (UR06, ST30) during the 2002-2003 period. Visually, the pulsotype A band pattern (Figure 1(a), lane 1) closely resembles to a community-associated MRSA clone reported in the South West Pacific region. This finding is similar to what was previously reported by Ribeiro et al. [38] in Porto Alegre, Brazil, and it could be in agreement to a regional dissemination of the Oceanìa Southwest Pacific Clone (OSPC).

Considering the PFGE results, inducible MLS_B_ phenotype, the presence of PVL genes and the CP serotype (Table 1), our findings suggest that several groups of CA-MRSA strains were circulating in Uruguay during the period of analysis. The major group (62 of the 90 strains) presented the following characteristics: pulsotype A, CP8 and PVL. Twenty-three of these 62 strains carried SCC*mec* type-IV. This group also included three NT strains but with positive PCR results for the *cp8 *gene (Table 1). These results might be complemented by multilocus sequence typing (MLST) or *spaA-*typing.

 In summary, three years after of the first finding of CA-MRSA isolates in Uruguay these strains are still producing SSTI, illustrating the stability over time of this emergent pathogen, as well as its excellent adaptation to the community enviroment. The cefoxitin disk test would be more reliable than the oxacillin disk test for the screening of the MRSA phenotype confered by the *mecA* gene and fusidic acid could be used as a topical empiric adjuvant treatment for nasal eradication of CA-MRSA. Our results also suggest that the presence of PVL genes appear as useful local markers for the detection of CA-MRSA strains. In this study we identified three major groups of CA-MRSA strains (1, 2, and 4) defined according to phenotypic and genotypic characteristics. The most frequent group, G1, showed a PFGE pattern identical to CA-MRSA strains previously isolated in Uruguay and Brazil.

## Figures and Tables

**Table 1:** Phenotypic and genotypic characteristics of CA-MRSA isolates recovered in Uruguay between 2004-2005. CP, capsular polysaccharide; iML_B_, inducible macrolide-lincosamide-streptogramin B resistance; PVL, Panton-Valentine leukocidin.

Group	PFGE type	CP	iMLS_B_ Phenotype	PVL	Number of strains
1	A	8	−	+	62^(a),(b)^
2	A, A1, A2	8	+	+	19
3	A3	8	−	−	1
4	B	8	+	+	4^(c)^
5	C	8	−	+	1
6	D	8	−	+	1
7	E	NT	+	−	1^(d)^
8	F	5	−	−	1

				Total	90

^(a)^Fifty-nine isolates showed positive result by colony immunoblot method with CP8 antibody and 3 strains showed positive result by PCR for the *cp8 *gene. ^(b)^Twenty-three strains showed SCC*mec* type-IV. ^(c)^All strains in this group showed SCC*mec* type-II. ^(d)^Positive PCR amplification for the *cp8 *gene.
